# Genetic correlations of alcohol consumption and alcohol use disorder with sex hormone levels in females and males

**DOI:** 10.3389/fpsyt.2025.1589688

**Published:** 2025-07-22

**Authors:** T. Cameron Waller, Ada M.-C. Ho, Anthony Batzler, Jennifer R. Geske, Victor M. Karpyak, Joanna M. Biernacka, Stacey J. Winham

**Affiliations:** ^1^ Department of Quantitative Health Sciences, Mayo Clinic, Rochester, MN, United States; ^2^ Department of Psychiatry and Psychology, Mayo Clinic, Rochester, MN, United States

**Keywords:** alcohol consumption, alcohol use disorder, testosterone, estradiol, SHBG, genetic correlation

## Abstract

**Background:**

Alcohol consumption behaviors and alcohol use disorder risk and presentation differ by sex, and are associated with blood concentrations of the steroid sex hormones, testosterone and estradiol, and their regulatory binding proteins, sex hormone binding globulin (SHBG) and albumin. Genetic variation is also associated with alcohol consumption, alcohol use disorder, and levels of these hormones and binding proteins.

**Methods:**

To assess the contribution of genetic factors to previously described phenotypic associations between alcohol-use traits and sex-hormone levels, we estimated genetic correlations (r_g_) using summary statistics from prior published, large sample size genome-wide association studies (GWAS) of alcohol consumption, alcohol dependence, testosterone, estradiol, SHBG, and albumin. We defined statistical significance at p < 0.005 and trends at p < 0.05.

**Results:**

For alcohol consumption, we observed positive genetic correlation (i.e. genetic effects in the same direction) with SHBG in females (r_g_ = 0.089, p = 0.004) and a trend toward negative genetic correlation (i.e. genetic effects in opposite directions) with bioavailable testosterone (r_g_ = -0.064, p = 0.032); however there were only trends toward positive genetic correlation with total testosterone in males (r_g_ = 0.084, p = 0.007) and with albumin in a sex-combined cohort (r_g_ = 0.082, p = 0.015). For alcohol dependence, we observed trends toward negative genetic correlation with total testosterone in females (r_g_ = -0.106, p = 0.024) and positive genetic correlation with BMI-adjusted SHBG in males (r_g_ = 0.119, p = 0.017). Some of these genetic correlations were different than the corresponding phenotypic associations, and some may suggest differences between females and males.

**Conclusions:**

Shared genetic effects might contribute to positive associations of alcohol consumption with albumin and between alcohol dependence and SHBG in males; however, most of the phenotypic associations between alcohol-use traits and levels of sex hormones and their binding proteins did not correspond to broadly shared genetic effects in the same direction. Some even corresponded to genetic effects in the opposite direction. Future studies of these traits should include GWAS on larger cohorts by sex and investigation of localized correlations of genetic effects and the relative contributions of heritable and environmental factors.

## 1 Introduction

Alcohol is the most commonly used and misused substance in the United States and represents a significant public health burden (1). Results from the 2023 National Survey on Drug Use and Health report that among individuals ages 12 and older, 16.8 million males (12.1%) and 12.0 million females (8.3%) are diagnosed with alcohol use disorder (2). The prevalence of alcohol use disorders differs by sex and gender in many populations around the world (3–6), although the gap is beginning to narrow due to increased alcohol consumption in females (7, 8). There are physiological differences by biological sex, such as the pharmacokinetics and pharmacodynamics (physiological response) of acute and chronic exposure to ethanol (9, 10). Acute intoxication also presents differently in females and males, with females having greater susceptibility to organ damage and other chronic health problems, such as hepatitis, hypertension, cardiomyopathy, diabetes, peripheral neuropathy, and volumetric brain loss, even at lower doses of ethanol proportional to body mass (5, 11). These sex differences may relate to the genomic composition of sex chromosomes in females (XX) and males (XY) and to endogenous concentrations of sex hormones (12), which are the major focus of this study. Beyond these physiological sex differences, complex social, cultural, and other environmental aspects of gender may also influence alcohol consumption behaviors (e.g. when, where, with whom, which types, and how much) and the onset, presentation, likelihood of seeking help, and recovery from addiction (5). Knowledge of the complex interactions between sex and gender and how these factors contribute to alcohol use behaviors and alcohol use disorders could inform preventive lifestyle decisions and actionable clinical interventions that improve patient prognosis.

Endogenous concentrations of the steroid sex hormones (e.g. estradiol, progesterone, and testosterone) differ between females and males, and these hormones regulate many aspects of physiology. Although testosterone and estradiol are mainly synthesized within the gonads, their regulatory influence extends far beyond the primary and secondary sex organs. Hence it is essential to maintain proper balance in the bioactivity of these hormones, and sequestration by the binding proteins sex hormone binding globulin (SHBG) (13) and albumin modulate the transport of hormones through the bloodstream and their accessibility to their respective receptors (14–16). The synthesis of both SHBG and albumin occurs primarily in the liver, which also bears the burden of eliminating most ethanol from the bloodstream (17); therefore, the liver’s function and susceptibility to damage constitutes one direct physiological link between alcohol use and the regulation of sex-hormone bioactivity. Furthermore, steroid sex hormones play a role in the activation of gene synthesis across many types of cells with diverse biological functions. The steroid sex hormones can regulate gene expression by binding to their respective intracellular receptors—the estrogen receptors (ER alpha and ER beta), the progesterone receptor (PR), and the androgen receptor (AR)—to establish hormone-receptor complexes which act as gene transcription factors. These hormone-receptor complexes then bind to specific DNA sequences known as hormone response elements that directly regulate the transcription of their target genes.

**Figure 1:**
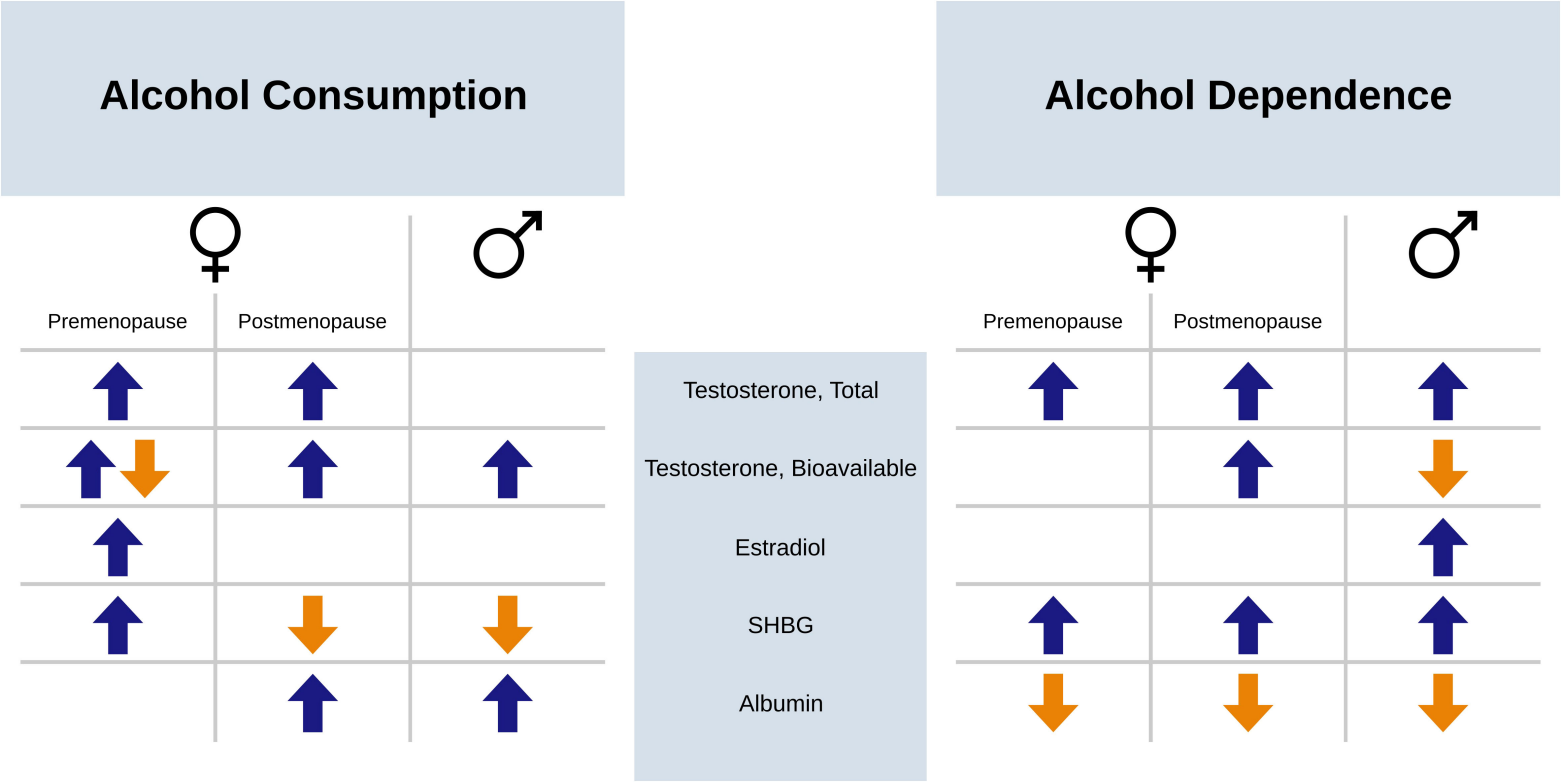
Graphical summary of prior observed associations between the phenotypes of alcohol consumption and alcohol dependence with sex hormones and their binding proteins. Positive associations are depicted with a blue upward arrow and negative associations are depicted with an orange downward arrow. Associations are shown separately for males (♂) and premenopause and postmenopause females (♀). SHBG, sex hormone binding globulin.

Evidence from prior studies involving large cohorts suggests that circulating levels of steroid sex hormones and their binding proteins share sex-specific associations with both alcohol consumption and alcohol use disorder (**Figure 1**). A general positive association has been found between alcohol consumption and testosterone in both males and females, including total, bioavailable, and free testosterone (18–20). Similarly, a general positive association has been reported between alcohol consumption and estradiol in females (18, 19, 21), particularly in premenopausal females during the luteal phase (19, 21). Lower SHBG levels have been associated with alcohol consumption in males (18, 20, 22), while findings in females were mixed (18, 19, 21). Higher albumin levels had been associated with alcohol consumption in both sexes (18, 23). Fewer studies have investigated associations between these hormones and proteins and AUD. Using UK Biobank data, we observed higher total testosterone and SHBG levels, but lower albumin levels, in both males and females with alcohol dependence history (18). Moreover, differences in estradiol levels between individuals with and without alcohol dependence history was observed only in males (18). These complex associations suggest that steroid sex hormones and their binding proteins may play an important role in the biological sex differences in alcohol use behaviors and consequences.

Beyond endogenous concentrations of sex hormones, genetics also contribute to the physiological differences of biological sex (12). Sex chromosome complement differs between females (XX) and males (XY), and several proteins with pervasive influence are encoded by genes on these sex chromosomes, such as prominent epigenetic and protein regulatory genes *KDM6A, KDM5C*, and *OGT* (12), as well as the androgen receptor (*AR*) (24). As noted above, concentrations of bioavailable steroid sex hormones bind and activate the AR or the estrogen receptors (ERalpha, ERbeta), which function as ligand-activated, nuclear-receptor transcription factors that regulate the expression of many genes. In fact, the transcriptional expression of roughly a third of all human genes across the genome, including the autosomes, differ by sex (25). Furthermore, levels of sex hormones are highly heritable in both females and males (26), and recent studies demonstrate that even the autosomal (chromosomes 1-22) genetic etiologies of testosterone, estradiol, and SHBG differ profoundly by sex (27–30). The genetic correlation between females and males, an estimate of shared genetic effects between the sexes, is near zero for testosterone (28–30), about 0.07 for estradiol (27, 30), and approximately 0.83% for SHBG (28–30), supporting distinct, sex-specific genetic etiology of these hormones, particularly for testosterone and estradiol.

Genetics also contribute to predisposition for alcohol-use traits, with heritability estimates of about 49% for alcohol use disorder (31, 32). Multiple genome-wide association studies (GWAS) have been conducted both for alcohol use behaviors (including frequency and quantity of alcohol consumption) and alcohol use disorders (including severe alcohol dependence) (33–43); however, it is unknown whether the genetic etiologies of these traits differ by sex (10). Previous GWAS on both alcohol consumption and alcohol use disorder in sex-stratified cohorts found no difference in the SNP heritability by sex, and the genetic correlation between females and males was not significantly less than one (38, 42–44). Analyses using sex-stratified GWAS for alcohol dependence were inconclusive, due to limitations of sample size in the female cohort (40, 44). While there is currently limited evidence of differences in the genetic etiologies of alcohol use traits between females and males (10), there are associations with the heritable predispositions of alcohol-use traits and sex hormone-related traits of female physiology. In females, greater alcohol consumption quantity and problematic alcohol use are genetically correlated with older age at menarche (33, 36, 38, 39, 42), younger age at menopause (35, 37), and reduced likelihood of bilateral oophorectomy (38). These alcohol-use traits are also genetically correlated with the use of oral contraception at a younger age (33, 34, 37, 38) and the lifetime use of hormonal therapy (33, 34, 37). Genetic correlation between alcohol use and these hormone-related traits in females, which may serve as surrogate markers of hormone levels, suggests that genetic factors may contribute to some of the aforementioned associations between alcohol-use and sex-hormone phenotypes.

Given the heritability of alcohol use behaviors, alcohol use disorders, and levels of steroid sex hormones and their binding proteins, together with the genetic relationships of alcohol use with multiple surrogate markers of sex hormone levels in females, it is likely that both heritable and environmental factors contribute to observed associations between alcohol-use and sex-hormone phenotypes. We hypothesized that alcohol-use traits share some aspects of their genetic etiology with steroid sex hormones and their binding proteins. We designed this study to quantify the broad, autosome-wide genetic similarity or dissimilarity between these traits. By utilizing publicly available summary statistics from previously published GWAS on alcohol-use and sex-hormone traits within large cohorts, we estimated genetic correlations between these traits and compared them to the previously published associations between corresponding phenotypes.

## 2 Methods

### 2.1 Summary statistics for large-cohort GWAS on alcohol use and sex hormones

We utilized summary statistics from previously published, large sample size GWAS of alcohol-use traits and blood concentrations of the steroid sex hormones to estimate the shared genetic etiology between these traits. For a description of the study selection strategy and identification of relevant GWAS summary statistics, see **Supplementary Methods**. **Supplementary Table S1** provides details about the summary statistics from these prior GWAS on quantity of alcohol consumption (39), risk of alcohol dependence (40), and blood concentrations of total testosterone (30), bioavailable testosterone (30), estradiol (27), SHBG (30), and albumin (45). **Supplementary Table S1** describes characteristics of each GWAS, including traits of interest, cohort sex, cohort ancestry, and sample size for each study (range N=46,568 to N=357,968). In some situations, the available GWAS summary statistics that were most appropriate for estimates of genetic correlation in this study differed slightly from the primary results reported in their respective publications (see **Supplementary Methods**). All sets of summary statistics included in our study came from GWAS or meta-analyses on cohorts of European ancestry. The GWAS on total testosterone (30), bioavailable testosterone (30), estradiol (27), and SHBG (30) used sex-stratified cohorts to accommodate major differences by sex in their physiological concentrations and genetic etiologies (27–30). In contrast, the GWAS on albumin (45) used a sex-combined cohort as this protein does not demonstrate major differences in concentration or genetic etiology between females and males (29). The GWAS on alcohol consumption quantity (39) and alcohol dependence (40) used sex-combined cohorts to maximize sample size, given that there is high genetic correlation between females and males in these traits.

### 2.2 Estimates of SNP heritability and genetic correlation

To assess the shared genetic etiologies of alcohol-use traits and sex-hormone levels, we estimated SNP heritabilities (h^2^) and genetic correlations (r_g_) using Linkage Disequilibrium (LD) Score Regression (LDSC) (46, 47). Using the summary statistics of GWAS from the traits of interest, LDSC estimates the SNP heritability (47) of one individual trait or the genetic correlation (46) between two traits, while accounting for LD between groups of SNPs (LD blocks). The SNP heritability represents the proportion of variance in one trait that is attributable to variation across common genotype SNPs, which is a fraction of the total heritability (the proportion of phenotype variance due to all genetic factors) (48, 49). In contrast, the genetic correlation between two traits is a broad measure of the similarity (r_g_ > 0) or dissimilarity (r_g_ < 0) in the direction and magnitude of their respective allelic effects (beta coefficients) across common genomic variants (SNPs).

In this study, we estimated genetic correlations between pairwise combinations of alcohol-use traits (alcohol consumption and alcohol dependence) and levels of sex hormones and their binding proteins (total testosterone, bioavailable testosterone, estradiol, SHBG, and albumin). We also compared these genetic correlations to the corresponding phenotypic associations previously reported (18) between alcohol-use traits and sex-hormone levels to offer insight about the contribution of genetic factors to the associations observed directly between these phenotypes. For example, if the direction of significant genetic correlation (r_g_ < 0; r_g_ > 0) between two traits matches the direction of association between corresponding phenotypes (beta < 0; beta > 0), the shared genetic effects may contribute to the observed phenotypic association. In contrast, if the genetic correlation is in the opposite direction of a phenotypic association, or if there is genetic correlation (r_g_ < 0; r_g_ > 0) in the absence of any apparent association between phenotypes (beta = 0), then non-genetic environmental factors might exert an opposing influence on the traits that masks or overpowers the genetic effects. Finally, a null genetic correlation (r_g_ = 0) is less conclusive, since this broad genome-wide measure cannot rule out the possibility that substantial shared genetic effects might still exist within more localized regions of the genome. For additional details on how estimates of SNP heritability and genetic correlation were obtained, see **Supplementary Methods**.

### 2.3 Statistical comparisons and correction for multiple testing

To adjust for multiple testing, we considered the number of biologically independent comparisons. Alcohol consumption and alcohol dependence are highly correlated, as are levels of the steroid sex hormones; in fact, bioavailable testosterone is directly calculated from the actual measured concentrations of total testosterone, SHBG, and albumin (15) such that concentrations of these biomolecules are mutually dependent. For all hormones and proteins except for albumin, we conducted analyses with stratification by sex to accommodate biologically relevant differences. Due to the extensive non-independence between these biomolecules and the alcohol-use traits, Bonferroni correction for the total number of comparisons (22 estimates of r_g_) would be inappropriate. Instead, we report 99% confidence intervals along with uncorrected p-values. We recommend a threshold of p < 0.005 for statistical significance, which is equivalent to Bonferroni correction for 10 independent comparisons. We also consider any genetic correlations with p < 0.05 as potential trends.

## 3 Results

To evaluate the extent to which shared genetic effects correspond to the previously reported associations between alcohol-use and sex-hormone traits, we estimated genetic correlations between prior-published GWAS summary statistics for the respective traits. **Supplementary Table S1** presents estimates of SNP heritability (h^2^) (see **Supplementary Results**). Genetic correlations (r_g_) of alcohol consumption (39) with the steroid sex hormones and their binding proteins are presented in **Table 1**, and genetic correlations with alcohol dependence (40) are presented in **Table 2**. For convenient comparison, **Tables 1**, **2** also present the phenotypic associations of the same alcohol-use and sex hormone traits from a recently published study using data from the UK Biobank (18), adjacent to the estimated genetic correlations.

**Table 1:** Genetic correlations and phenotypic associations of alcohol consumption with steroid sex hormones and their binding proteins.

Secondary Trait:	Sex	Primary Trait: Alcohol Consumption						
		Genetic Correlation					Phenotype Association^1^	
		r_g_	se	99%ci low	99%ci high	p	beta	p
Testosterone, Total	Female	0.0132	0.0311	-0.0669	0.0933	0.6704	**0.019**	**< 0.001**
Testosterone, Total	Male	0.0837	0.0309	0.0041	0.1633	0.0068	-0.001	0.645
Testosterone, Bioavailable	Female	-0.0642	0.0299	-0.1412	0.0128	0.0317	**0.031**	**< 0.001**
Testosterone, Bioavailable	Male	0.0591	0.0343	-0.0293	0.1475	0.0844	**0.024**	**< 0.001**
Estradiol	Female	-0.1099	0.1158	-0.4082	0.1884	0.3427	**0.009**	**0.002**
Estradiol	Male	-0.0094	0.0650	-0.1768	0.1580	0.8852	-0.004	0.430
SHBG	Female	**0.0894**	**0.0308**	**0.0101**	**0.1687**	**0.0037**	NA	NA
SHBG	Male	0.0560	0.0326	-0.0280	0.1400	0.0859	NA	NA
SHBG-BMI	Female	0.0271	0.0323	-0.0561	0.1103	0.4013	**-0.023**	**< 0.001**
SHBG-BMI	Male	0.0343	0.0344	-0.0543	0.1229	0.3176	**-0.030**	**< 0.001**
Albumin	All	0.0819	0.0337	-0.0049	0.1687	0.0150	NA	NA
Albumin	Female	NA	NA	NA	NA	NA	**0.054**	**< 0.001**
Albumin	Male	NA	NA	NA	NA	NA	**0.030**	**< 0.001**

We used prior published GWAS summary statistics and linkage disequilibrium score regression (LDSC) to estimate genetic correlations of alcohol consumption with steroid sex hormones and their binding proteins (**Supplementary Table S1**), and we compared these novel genetic correlations to prior published associations between phenotypes corresponding to these traits. **Supplementary Table S1** presents further details on the respective sets of GWAS summary statistics that we used in this study. Effect coefficients (beta) from phenotypic associations are not on the same scale as genetic correlations (r_g_). Bold values indicate statistically significant associations.

SHBG, sex hormone binding globulin; BMI, body mass index; SHBG-BMI, designation of GWAS on SHBG with adjustment for BMI as a covariate; GWAS, genome-wide association study; r_g_, genetic correlation; se, standard error; 99%ci low, lower limit of 99% confidence interval; 99%ci high, upper limit of 99% confidence interval; p, p-value for testing the null hypothesis that genetic correlation (r_g_) or phenotypic association (beta) is zero; NA, not available.

1: Prior published phenotypic associations from Ho et al. (18).

**Table 2:** Genetic correlations and phenotypic associations of alcohol dependence with steroid sex hormones and their binding proteins.

Secondary trait:	Sex	Primary trait: Alcohol dependence						
		Genetic correlation					Phenotype association^1^	
		r_g_	se	99%ci low	99%ci high	p	beta	p
Testosterone, Total	Female	-0.1058	0.0470	-0.2269	0.0153	0.0243	**0.211**	**< 0.001**
Testosterone, Total	Male	0.0420	0.0513	-0.0901	0.1741	0.4133	**0.225**	**< 0.001**
Testosterone, Bioavailable	Female	-0.0528	0.0521	-0.1870	0.0814	0.3105	**0.108**	**0.046**
Testosterone, Bioavailable	Male	-0.0362	0.0567	-0.1823	0.1099	0.5230	**-0.078**	**< 0.001**
Estradiol	Female	-0.1827	0.1913	-0.6755	0.3101	0.3397	-0.061	0.474
Estradiol	Male	0.1594	0.1203	-0.1505	0.4693	0.1849	**0.257**	**< 0.001**
SHBG	Female	-0.0206	0.0537	-0.1589	0.1177	0.7006	NA	NA
SHBG	Male	0.0670	0.0500	-0.0618	0.1958	0.1802	NA	NA
SHBG-BMI	Female	0.0258	0.0509	-0.1053	0.1569	0.6128	**0.124**	**0.002**
SHBG-BMI	Male	0.1185	0.0498	-0.0098	0.2468	0.0174	**0.430**	**< 0.001**
Albumin	All	0.0054	0.0484	-0.1193	0.1301	0.9110	NA	NA
Albumin	Female	NA	NA	NA	NA	NA	**-0196**	**< 0.001**
Albumin	Male	NA	NA	NA	NA	NA	**-0.270**	**< 0.001**

We used prior published GWAS summary statistics and linkage disequilibrium score regression (LDSC) to estimate genetic correlations of alcohol dependence with steroid sex hormones and their binding proteins (**Supplementary Table S1**), and we compared these novel genetic correlations to prior published associations between phenotypes corresponding to these traits. **Supplementary Table S1** presents further details on the respective sets of GWAS summary statistics that we used in this study. Effect coefficients (beta) from phenotypic associations are not on the same scale as genetic correlations (r_g_). Bold values indicate statistically significant associations.

SHBG, sex hormone binding globulin; BMI, body mass index; SHBG-BMI, designation of GWAS on SHBG with adjustment for BMI as a covariate; GWAS, genome-wide association study; r_g_, genetic correlation; se, standard error; 99%ci low, lower limit of 99% confidence interval; 99%ci high, upper limit of 99% confidence interval; p, p-value for testing the null hypothesis that genetic correlation (r_g_) or phenotypic association (beta) is zero; NA, not available.

1: Prior published phenotypic associations from Ho et al. (18).

### 3.1 Genetic correlations of alcohol consumption with the steroid sex hormones and their binding proteins

**Figure 2:**
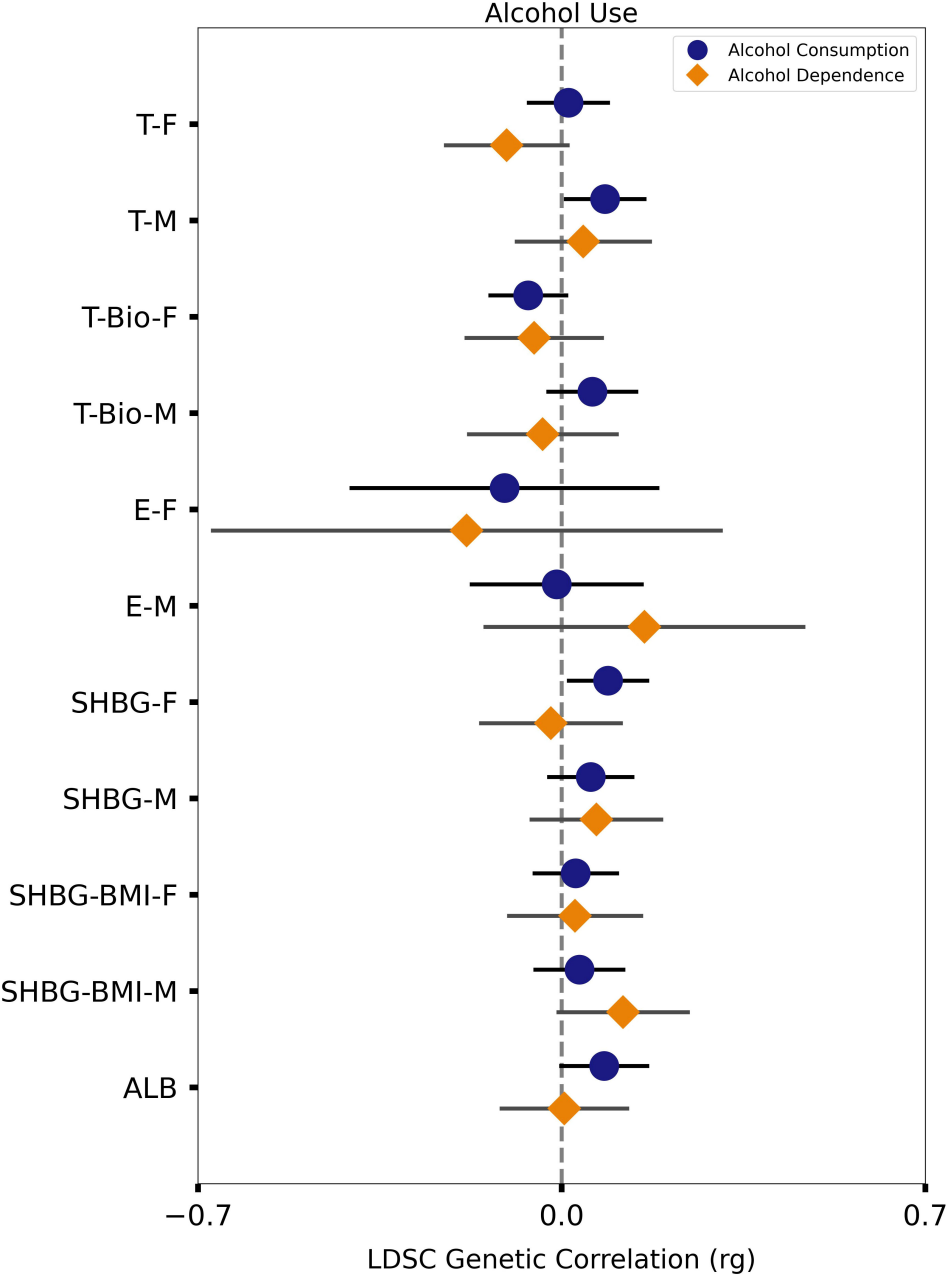
Genetic correlations of steroid sex hormones and their binding proteins with alcohol consumption and alcohol dependence. Estimates of genetic correlation (r_g_) are plotted along with their 99% confidence intervals (horizontal X axis) for alcohol consumption (blue circle) and alcohol dependence (orange diamond) and for each hormone or protein, separately in females and males (vertical Y axis). Values correspond to those in **Tables 1**, **2**. F, female; M, male; T, total testosterone; T-Bio, bioavailable testosterone; E, estradiol; SHBG, sex hormone binding globulin; SHBG-BMI, sex hormone binding globulin with BMI adjustment; ALB, albumin.

In males, alcohol consumption trended toward positive genetic correlation with total testosterone in males (r_g_ = 0.08, p = 0.007), but not with bioavailable testosterone (r_g_ = 0.06, p = 0.08) (**Table 1**). In females, alcohol consumption was not genetically correlated with total testosterone (r_g_ = 0.01, p = 0.67), but trended toward negative genetic correlation with bioavailable testosterone (r_g_ = -0.06, p = 0.03) (**Table 1**). There was no significant genetic correlation of alcohol consumption with estradiol in females (r_g_ = -0.11, p = 0.34) or males (r_g_ = -0.009, p = 0.89) (**Table 1**), although standard errors were large and confidence intervals were wide. Alcohol consumption was positively genetically correlated with SHBG in females (r_g_ = 0.09, p = 0.004), but not with SHBG in males (r_g_ = 0.06, p = 0.09) (**Table 1**); however, there was no genetic correlation when the GWAS of SHBG adjusted for BMI in females (r_g_ = 0.03, p = 0.40) and males (r_g_ = 0.03, p = 0.32) (**Table 1**). Alcohol consumption also trended toward positive genetic correlation with albumin in both sexes combined (r_g_ = 0.08, p = 0.02) (**Table 1**). Overall, alcohol consumption was significantly positively genetically correlated with SHBG only in females, but there were trends towards positive genetic correlation with total testosterone in males and albumin in both sexes, and trends towards negative genetic correlation with bioavailable testosterone in females (**Figure 2**).

### 3.2 Genetic correlations of alcohol dependence with the steroid sex hormones and their binding proteins

Alcohol dependence trended toward negative genetic correlation with total testosterone in females (r_g_ = -0.11, p = 0.02), but not males (r_g_ = 0.04, p = 0.41); bioavailable testosterone was not genetically correlated with alcohol dependence in females (r_g_ = -0.05, p = 0.31) or males (r_g_ = -0.04, p = 0.52) (**Table 2**). There was no significant genetic correlation of alcohol dependence with estradiol in females (r_g_ = -0.18, p = 0.34) or males (r_g_ = 0.16, p = 0.18) (**Table 2**), although these estimates had large standard errors and wide confidence intervals. There was no genetic correlation of alcohol dependence with SHBG in females (r_g_ = -0.02, p = 0.70) or males (r_g_ = 0.07, p = 0.18); however, alcohol dependence trended toward positive genetic correlation with BMI-adjusted SHBG in males (r_g_ = 0.12, p = 0.02) but not females (r_g_ = 0.03, p = 0.61) (**Table 2**). Alcohol dependence was not genetically correlated with albumin in males and females (r_g_ = 0.005, p = 0.91) (**Table 2**). Overall, no significant genetic correlations were observed for alcohol dependence; however, there were trends towards negative genetic correlation with total testosterone in females and positive genetic correlation with BMI-adjusted SHBG in males (**Figure 2**).

## 4 Discussion

This study aimed to assess the impact of broadly shared genetic etiologies on the relationship between alcohol-use traits and sex-hormone levels. This study builds on multiple previous studies that have observed associations of acute or chronic alcohol use behaviors (including frequency and quantity of consumption) and alcohol use disorders (including dependence) with blood concentrations of steroid sex hormones and their regulatory binding proteins (18–23, 50, 51), including our recent study that showed striking differences by sex in associations of alcohol consumption and alcohol dependence with testosterone, estradiol, SHBG, and albumin in data from the UK Biobank (18). In this study, we hypothesized that both environmental and heritable factors contribute to these associations between phenotypes. To test this hypothesis, we estimated the genetic correlations of alcohol-use traits with sex-hormone levels using prior GWAS results. We found that alcohol consumption was positively genetically correlated with SHBG in females and also trended toward positive genetic correlation with total testosterone in males, negative genetic correlation with bioavailable testosterone in females, and positive genetic correlation with albumin in both sexes. We also found that alcohol dependence trended toward negative genetic correlation with total testosterone in females and also trended toward positive genetic correlation with SHBG in males, but only when the GWAS of SHBG was adjusted for BMI. Collectively, these findings suggest that sex-specific heritable predispositions for higher or lower levels of some steroid sex hormones or their binding proteins might contribute to the etiologies of alcohol-use behaviors and disorders (52).

When compared to previously reported phenotype associations of hormone levels with alcohol consumption and dependence, we observed two instances in which the direction of genetic correlation was consistent (18). The trend toward positive genetic correlation of alcohol consumption with albumin in a sex-combined cohort paralleled the previously published positive phenotypic association. Additionally, alcohol dependence was positively associated with BMI-adjusted levels of SHBG and similarly trended toward positive genetic correlation with BMI-adjusted SHBG in males. In these instances, the shared positive genetic effects may contribute to the positive associations observed between phenotypes.

However, other genetic correlations of alcohol traits with steroid sex hormones and their binding proteins were inconsistent with the corresponding associations between phenotypes, with either null association or associations in opposite directions (18). Specifically, alcohol consumption trended toward positive genetic correlation with total testosterone in males but not in females, even though the corresponding phenotypes were positively associated in females but not in males. While alcohol consumption associated positively with bioavailable testosterone levels in both sexes, the corresponding trend toward genetic correlation was negative in females, unlike males. Alcohol consumption was positively associated with estradiol in females, but genetic correlations with estradiol were null in both males and females. Levels of BMI-adjusted SHBG were negatively associated with alcohol consumption in both sexes, but the only trend toward genetic correlation in either sex was positive and without adjustment for BMI. In contrast to alcohol consumption, alcohol dependence shared a different pattern of phenotypic associations (18) and genetic correlations with steroid sex hormones and their binding proteins. Total testosterone trended toward negative genetic correlation with alcohol dependence in females, despite positive associations between the corresponding phenotypes in both sexes. While alcohol dependence was positively associated with levels of bioavailable testosterone in females and negatively associated in males, the genetic correlations were null. Similarly, alcohol dependence and estradiol were not genetically correlated in either sex, even though there was a positive association between corresponding phenotypes in males. Levels of BMI-adjusted SHBG were positively associated with alcohol dependence in both sexes, although the genetic correlation was null in females. Levels of albumin were negatively associated with alcohol dependence, but the corresponding genetic correlation was null.

Overall, we found that most of the prior published associations (18) between alcohol-use traits and sex-hormone levels did not correspond to genetic correlations with consistent directionality. Specifically, while many of the phenotype associations were statistically significant, many of the genetic correlations were null. It is important to acknowledge that because the genetic correlation is a broad, autosome-wide measure of shared genetic effects, null estimates of genetic correlation cannot dismiss the possibility of narrower, region-specific pleiotropy, and it is possible that more localized genomic regions may play a role in these relationships. However, the instances of genetic correlation in the absence of phenotypic association or in an opposite direction may also suggest that complex environmental factors might mask or even overpower the influence of shared heritable factors between some of these alcohol-use and sex-hormone traits. Further research is needed to better explore both localized genetic effects, as well as the role of gene-environment interactions.

The example of SHBG illustrates the complexity of studying genetic etiology in the context of dynamic regulation and environmental factors. This high-affinity binding protein with tightly regulated and dynamic synthesis in the alcohol-sensitive liver has an important role in the relationship between alcohol use and the steroid sex hormones, especially in males (18). Previously, we showed that SHBG mediated the associations of total testosterone with alcohol consumption and alcohol use disorder in both males and females, but it acted as a moderator of these same associations only in males (18). In this study, we observed major differences in the genetic correlation of SHBG with both alcohol consumption and alcohol dependence that depended on adjustment for BMI in the GWAS of SHBG. Notably, higher BMI is associated with lower concentrations of SHBG in the blood (53, 54); however, it is not general adiposity (subcutaneous or visceral) that drives this association (53). Rather, obesity and excessive alcohol consumption both can cause damage to the liver (55) in the form of fatty liver disease and steatohepatitis, and this fat accumulation and inflammation of the liver might in turn decrease synthesis of SHBG (53). As higher BMI is also associated with higher alcohol consumption (56), BMI may lead to collider bias (57) on the association between SHBG and alcohol use. Therefore, interpretation of the results from GWAS with or without BMI adjustment is complex, as acknowledged by the authors who published the GWAS of SHBG (30). Thus, we chose to report genetic correlations for SHBG both with and without adjustment for BMI, even though the SHBG GWAS that adjusted for BMI showed greater SNP heritability (30).

## 5 Limitations

Strengths of this study include the use of previously published, publicly available GWAS with large sample sizes, rigorous application of LDSC regression to assess SNP heritability and genetic correlation, and a distinction between the traits of alcohol consumption and alcohol dependence. However, several limitations also need to be acknowledged. First, because this study relies on the findings from previously published studies, there is inherent variability in study design and methodology across GWAS, such as differences in target populations as well as ascertainment and data collection methods, which may limit the generalizability of findings in this study. In particular, the prior GWAS were all cross-sectional studies that adjusted for different covariates across the alcohol and hormone traits. Complex sociocultural and other environmental factors contribute to alcohol use behaviors (5), and it is also difficult for cross-sectional, single-time-point measurements of steroid sex hormones and their binding proteins to adequately represent the dynamic concentrations that fluctuate within individuals on diurnal, monthly, and seasonal rhythms with influence from many other factors, especially in females. Second, GWAS of sex-hormone levels may not have adjusted for all relevant covariates, particularly in females. Specifically, the GWAS (30) for total testosterone did not adjust for aspects of female physiology such as phase of the menstrual cycle, menopause, or use of oral contraception and other hormonal therapies, whereas the GWAS for bioavailable testosterone (30) and for estradiol (27) adjusted for some of these covariates. Furthermore, the GWAS of testosterone and estradiol were not stratified by menopause status in females, which correspond to major differences in the concentrations of these steroid sex hormones. Whereas previous studies have performed GWAS of testosterone and estradiol separately in premenopause and postmenopause females (29, 58), to our knowledge the summary statistics are not publicly available. Third, the available GWAS summary statistics for steroid sex hormones and their binding proteins did not capture genetic etiologies earlier than age 40. These GWAS used data from the UK Biobank (59), with a minimum age at the time of enrollment of approximately 40 years (60), limiting the generalizability of findings to younger populations that may be important for the alcohol-related traits. Fourth, estimates of SNP heritability and genetic correlation for estradiol had low precision, partly related to the older age of the GWAS cohort. While the SNP heritabilities were high for total testosterone, bioavailable testosterone, SHBG, and albumin, SNP heritability estimates for estradiol were much lower with large standard errors. Notably, the biochemical assay used to measure concentrations of estradiol in UK Biobank samples lacked sensitivity (lower limit of detection) (61), and investigators accommodated the high rates of missingness in these measurements by performing GWAS on a dichotomous variable for the detectability of estradiol (27, 30), which reduced precision of the measured estradiol levels. The lower precision in estimates of SNP heritability and genetic correlation for estradiol may have obscured any shared genetic architecture with alcohol-use traits.

Fifth, many of the observed genetic correlations were null, which are less conclusive, as null genetic correlations cannot rule out the potential for localized genetic overlap, which could be further investigated through fine-mapping or regional LD analyses.

This study assessed the shared genetic etiologies between alcohol-use traits and sex-hormone levels across the autosomes, which leaves an important knowledge gap for future investigation, given the relevance of chromosomes X and Y to sex differences of many traits. Historically, the majority of GWAS have ignored the sex chromosomes, which continues to persist today. A recent review estimated that of all publicly available GWAS summary statistics, only 25% included variants on chromosome X and only 3% included variants on chromosome Y (62). The sex chromosomes present unique features compared to the autosomes, including sex-dependent diploid or haploid dosages of X, recombination only within the pseudo-autosomal region of Y, and the developmental mosaic of X-inactivation in females (62), which lead to several analytical challenges that are beginning to be addressed (63). However, many analytical methods avoid these challenges by ignoring the sex chromosomes, including currently available methods to estimate SNP heritability and genetic correlation, such as LDSC (46, 47). The genetic etiologies of testosterone, estradiol, and SHBG all differ quantitatively between females and males even when only considering the autosomes (27–30), but prior GWAS have demonstrated additional genome-wide-significant associations with variants on the X chromosome (27, 30). Furthermore, for the alcohol use traits, the recent meta-analysis GWAS on alcohol consumption and alcohol use disorder excluded the sex chromosomes (33, 39, 40). Due to the exclusion of chromosomes X and Y, the estimates of SNP heritability reported here may be artificially low, and it is possible that the genetic etiologies of alcohol use and sex hormones share additional pleiotropy across variants on the sex chromosomes. Indeed, a previous study found that variants on the X chromosome contributed to sex differences in multiple complex heritable diseases (64), and another study found evidence that sex-specific associations across autosomal variants alone were unlikely to explain the strong sex differences in several psychiatric traits (44). Future GWAS studies should include both the X and Y chromosomes in analysis and in public release of GWAS summary statistics, and software modifications should be made to include the results of the X and Y chromosomes in estimates of SNP heritability and genetic correlation. The primary analytical challenges occur when males and females are analyzed together, and performing sex-stratified analyses can avoid many of these challenges. For example, in females, the X chromosome could be modeled in the same manner as the diploid autosomes, and in males, the X and Y chromosome haploid genotypes could be converted to a diploid scale.

## 6 Conclusions and future directions

This study found that some effects of genetic variation on alcohol-use traits may be shared with the effects of genetic variation on levels of sex-hormones and their binding proteins. Patterns of genetic correlation of sex hormones with alcohol consumption differed from those with alcohol dependence, emphasizing the distinction between these alcohol-use traits. Shared genetic effects may contribute to the positive relationship (18) between alcohol consumption and levels of albumin in both sexes, and between alcohol dependence and levels of BMI-adjusted SHBG in males. Other genetic correlations differed from associations between corresponding phenotypes, which may indicate distinct genetic etiology between these hormones and alcohol consumption or dependence. However, some of the null genetic correlations may represent false negative findings, and further investigation is needed. Future studies should include the latest GWAS on alcohol use traits with larger sample sizes (33, 65), some of which stratified by sex (66). There is a need for GWAS on higher-quality measurements of sex-hormone levels in large cohorts, especially for estradiol, which stratify by sex, represent younger and older ages, and adjust for hormone-influencing factors, and for all GWAS to include the X and Y chromosomes. Future studies would also benefit from use of methods (52, 67–72) that offer better resolution or statistical power to detect pleiotropy either genome-wide or within narrower genomic regions, development of methods to include the X and Y chromosomes, and further evaluation of the complex interactions of heritable and environmental (52) factors, including sociocultural factors related to gender (5). Collectively, these future studies will be important to describe the relevance of steroid sex hormones and their binding proteins to differences by sex and gender in alcohol use behaviors and alcohol use disorders. Alcohol use disorder remains a significant clinical and public health concern for growing numbers of men and women, with different behavioral patterns and clinical presentation by sex. A better understanding of the interplay between genetics, sex hormones, and alcohol use could inform preventive lifestyle decisions and actionable clinical interventions that improve patient prognosis for both men and women.

## Data Availability

This study applied prior published and publicly available computational software tools to process and analyze publicly available summary statistics from prior published genome-wide association studies (GWAS). Software tools and reference data are accessible from sources cited in the Methods and **Supplementary Methods**. Detailed information about the prior published GWAS is available from original publications cited in **Supplementary Table S1**, and the summary statistics are accessible from online repositories including the Psychiatric Genomics Consortium (https://pgc.unc.edu/), the GWAS & Sequencing Consortium of Alcohol and Nicotine use (GSCAN) at the University of Minnesota (https://genome.psych.umn.edu/index.php/GSCAN), the National Human Genome Research Institute (NHGRI) and European Bioinformatics Institute (EBI) GWAS Catalog (https://www.ebi.ac.uk/gwas/), and the Zenodo archive (https://zenodo.org).

## References

[1] Cohen SM Alexander RS Holt SR. The spectrum of alcohol use: epidemiology, diagnosis, and treatment. Med Clin North Am. (2022) 106:43–60. doi: 10.1016/j.mcna.2021.08.003, PMID: 34823734

[2] Results from the 2023 National Survey on Drug Use and Health (Nsduh): Key Substance Use and Mental Health Indicators in the United States. Substance Abuse and Mental Health Services Administration (Samhsa). Available at: https://Library.Samhsa.Gov/Product/2023-Nsduh-Report/Pep24-07-021 Available at: https://www.samhsa.gov/data/sites/default/files/reports/rpt47095/National%20Report/National%20Report/2023-nsduh-annual-national.htm.

[3] Collaborators GBDA. Population-level risks of alcohol consumption by amount, geography, age, sex, and year: A systematic analysis for the global burden of disease study 2020. Lancet. (2022) 400:185–235. doi: 10.1016/S0140-6736(22)00847-9, PMID: 35843246 PMC9289789

[4] Collaborators GBDA. Alcohol use and burden for 195 countries and territories, 1990-2016: A systematic analysis for the global burden of disease study 2016. Lancet. (2018) 392:1015–35. doi: 10.1016/S0140-6736(18)31310-2, PMID: 30146330 PMC6148333

[5] Erol A Karpyak VM. Sex and gender-related differences in alcohol use and its consequences: contemporary knowledge and future research considerations. Drug Alcohol Depend. (2015) 156:1–13. doi: 10.1016/j.drugalcdep.2015.08.023, PMID: 26371405

[6] Gowing LR Ali RL Allsop S Marsden J Turf EE West R. Global statistics on addictive behaviours: 2014 status report. Addiction. (2015) 110:904–19. doi: 10.1111/add.12899, PMID: 25963869

[7] White AM. Gender differences in the epidemiology of alcohol use and related harms in the United States. Alcohol Res. (2020) 40:1. doi: 10.35946/arcr.v40.2.01, PMID: 33133878 PMC7590834

[8] Keyes KM Jager J Mal-Sarkar T Patrick ME Rutherford C Hasin D. Is there a recent epidemic of women’s drinking? A critical review of national studies. Alcohol Clin Exp Res. (2019) 43:1344–59. doi: 10.1111/acer.14082, PMID: 31074877 PMC6602861

[9] Vatsalya V Byrd ND Stangl BL Momenan R Ramchandani VA. Influence of age and sex on alcohol pharmacokinetics and subjective pharmacodynamic responses following intravenous alcohol exposure in humans. Alcohol. (2023) 107:144–52. doi: 10.1016/j.alcohol.2022.08.010, PMID: 36152778 PMC10023287

[10] Hitzemann R Bergeson SE Berman AE Bubier JA Chesler EJ Finn DA. Sex differences in the brain transcriptome related to alcohol effects and alcohol use disorder. Biol Psychiatry. (2022) 91:43–52. doi: 10.1016/j.biopsych.2021.04.016, PMID: 34274109 PMC8558111

[11] Bradley KA Badrinath S Bush K Boyd-Wickizer J Anawalt B. Medical risks for women who drink alcohol. J Gen Intern Med. (1998) 13:627–39. doi: 10.1046/j.1525-1497.1998.cr187.x, PMID: 9754520 PMC1497016

[12] Arnold AP. X chromosome agents of sexual differentiation. Nat Rev Endocrinol. (2022) 18:574–83. doi: 10.1038/s41574-022-00697-0, PMID: 35705742 PMC9901281

[13] Laurent MR Hammond GL Blokland M Jardi F Antonio L Dubois V. Sex hormone-binding globulin regulation of androgen bioactivity *in vivo*: validation of the free hormone hypothesis. Sci Rep. (2016) 6:35539. doi: 10.1038/srep35539, PMID: 27748448 PMC5066276

[14] Hammond GL. Access of reproductive steroids to target tissues. Obstet Gynecol Clin North Am. (2002) 29:411–23. doi: 10.1016/s0889-8545(02)00008-6, PMID: 12353665

[15] Sodergard R Backstrom T Shanbhag V Carstensen H. Calculation of free and bound fractions of testosterone and estradiol-17 beta to human plasma proteins at body temperature. J Steroid Biochem. (1982) 16:801–10. doi: 10.1016/0022-4731(82)90038-3, PMID: 7202083

[16] Dunn JF Nisula BC Rodbard D. Transport of steroid hormones: binding of 21 endogenous steroids to both testosterone-binding globulin and corticosteroid-binding globulin in human plasma. J Clin Endocrinol Metab. (1981) 53:58–68. doi: 10.1210/jcem-53-1-58, PMID: 7195404

[17] Hyun J Han J Lee C Yoon M Jung Y. Pathophysiological aspects of alcohol metabolism in the liver. Int J Mol Sci. (2021) 22(11):5717. doi: 10.3390/ijms22115717, PMID: 34071962 PMC8197869

[18] Ho AM Pozsonyiova S Waller TC Song Y Geske JR Karpyak VM. Associations of sex-related steroid hormones and proteins with alcohol dependence: A United Kingdom biobank study. Drug Alcohol Depend. (2023) 244:109781. doi: 10.1016/j.drugalcdep.2023.109781, PMID: 36701934 PMC10168535

[19] Tin Tin S Key TJ Reeves GK. Alcohol intake and endogenous hormones in pre- and postmenopausal women: findings from the Uk biobank. Cancer Epidemiol Biomarkers Prev. (2021) 30:2294–301. doi: 10.1158/1055-9965.EPI-21-0789, PMID: 34607837 PMC9398104

[20] Hansen ML Thulstrup AM Bonde JP Olsen J Hakonsen LB Ramlau-Hansen CH. Does last week’s alcohol intake affect semen quality or reproductive hormones? A cross-sectional study among healthy young Danish men. Reprod Toxicol. (2012) 34:457–62. doi: 10.1016/j.reprotox.2012.06.004, PMID: 22732148

[21] Hirko KA Spiegelman D Willett WC Hankinson SE Eliassen AH. Alcohol consumption in relation to plasma sex hormones, prolactin, and sex hormone-binding globulin in premenopausal women. Cancer Epidemiol Biomarkers Prev. (2014) 23:2943–53. doi: 10.1158/1055-9965.EPI-14-0982, PMID: 25281368 PMC4257878

[22] Jensen TK Gottschau M Madsen JO Andersson AM Lassen TH Skakkebaek NE. Habitual alcohol consumption associated with reduced semen quality and changes in reproductive hormones; a cross-sectional study among 1221 young Danish men. BMJ Open. (2014) 4:e005462. doi: 10.1136/bmjopen-2014-005462, PMID: 25277121 PMC4185337

[23] Alatalo P Koivisto H Puukka K Hietala J Anttila P Bloigu R. Biomarkers of liver status in heavy drinkers, moderate drinkers and abstainers. Alcohol Alcohol. (2009) 44:199–203. doi: 10.1093/alcalc/agn099, PMID: 19054785

[24] Ross MT Grafham DV Coffey AJ Scherer S McLay K Muzny D. The DNA sequence of the human X chromosome. Nature. (2005) 434:325–37. doi: 10.1038/nature03440, PMID: 15772651 PMC2665286

[25] Oliva M Munoz-Aguirre M Kim-Hellmuth S Wucher V Gewirtz ADH Cotter DJ. The impact of sex on gene expression across human tissues. Science. (2020) 369(6509):eaba3006. doi: 10.1126/science.aba3066, PMID: 32913072 PMC8136152

[26] Grotzinger AD Mann FD Patterson MW Herzhoff K Tackett JL Tucker-Drob EM. Twin models of environmental and genetic influences on pubertal development, salivary testosterone, and estradiol in adolescence. Clin Endocrinol (Oxf). (2018) 88:243–50. doi: 10.1111/cen.13522, PMID: 29161770 PMC5771835

[27] Schmitz D Ek WE Berggren E Hoglund J Karlsson T Johansson A. Genome-wide association study of estradiol levels and the causal effect of estradiol on bone mineral density. J Clin Endocrinol Metab. (2021) 106:e4471–e86. doi: 10.1210/clinem/dgab507, PMID: 34255042 PMC8530739

[28] Sinnott-Armstrong N Naqvi S Rivas M Pritchard JK. Gwas of three molecular traits highlights core genes and pathways alongside a highly polygenic background. Elife. (2021) 10:e58615. doi: 10.7554/eLife.58615, PMID: 33587031 PMC7884075

[29] Flynn E Tanigawa Y Rodriguez F Altman RB Sinnott-Armstrong N Rivas MA. Sex-specific genetic effects across biomarkers. Eur J Hum Genet. (2021) 29:154–63. doi: 10.1038/s41431-020-00712-w, PMID: 32873964 PMC7794464

[30] Ruth KS Day FR Tyrrell J Thompson DJ Wood AR Mahajan A. Using human genetics to understand the disease impacts of testosterone in men and women. Nat Med. (2020) 26:252–8. doi: 10.1038/s41591-020-0751-5, PMID: 32042192 PMC7025895

[31] Verhulst B Neale MC Kendler KS. The heritability of alcohol use disorders: A meta-analysis of twin and adoption studies. Psychol Med. (2015) 45:1061–72. doi: 10.1017/S0033291714002165, PMID: 25171596 PMC4345133

[32] Walters GD. The heritability of alcohol abuse and dependence: A meta-analysis of behavior genetic research. Am J Drug Alcohol Abuse. (2002) 28:557–84. doi: 10.1081/ada-120006742, PMID: 12211366

[33] Saunders GRB Wang X Chen F Jang SK Liu M Wang C. Genetic diversity fuels gene discovery for tobacco and alcohol use. Nature. (2022) 612:720–4. doi: 10.1038/s41586-022-05477-4, PMID: 36477530 PMC9771818

[34] Zhou H Sealock JM Sanchez-Roige S Clarke TK Levey DF Cheng Z. Genome-wide meta-analysis of problematic alcohol use in 435,563 individuals yields insights into biology and relationships with other traits. Nat Neurosci. (2020) 23:809–18. doi: 10.1038/s41593-020-0643-5, PMID: 32451486 PMC7485556

[35] Thompson A Cook J Choquet H Jorgenson E Yin J Kinnunen T. Functional validity, role, and implications of heavy alcohol consumption genetic loci. Sci Adv. (2020) 6:eaay5034. doi: 10.1126/sciadv.aay5034, PMID: 31998841 PMC6962045

[36] Evangelou E Gao H Chu C Ntritsos G Blakeley P Butts AR. New alcohol-related genes suggest shared genetic mechanisms with neuropsychiatric disorders. Nat Hum Behav. (2019) 3:950–61. doi: 10.1038/s41562-019-0653-z, PMID: 31358974 PMC7711277

[37] Gelernter J Sun N Polimanti R Pietrzak RH Levey DF Lu Q. Genome-wide association study of maximum habitual alcohol intake in >140,000 U.S. European and african american veterans yields novel risk loci. Biol Psychiatry. (2019) 86:365–76. doi: 10.1016/j.biopsych.2019.03.984, PMID: 31151762 PMC6919570

[38] Kranzler HR Zhou H Kember RL Vickers Smith R Justice AC Damrauer S. Genome-wide association study of alcohol consumption and use disorder in 274,424 individuals from multiple populations. Nat Commun. (2019) 10:1499. doi: 10.1038/s41467-019-09480-8, PMID: 30940813 PMC6445072

[39] Liu M Jiang Y Wedow R Li Y Brazel DM Chen F. Association Studies of up to 1.2 Million Individuals Yield New Insights into the Genetic Etiology of Tobacco and Alcohol Use. Nat Genet. (2019) 51:237–44. doi: 10.1038/s41588-018-0307-5, PMID: 30643251 PMC6358542

[40] Walters RK Polimanti R Johnson EC McClintick JN Adams MJ Adkins AE. Transancestral gwas of alcohol dependence reveals common genetic underpinnings with psychiatric disorders. Nat Neurosci. (2018) 21:1656–69. doi: 10.1038/s41593-018-0275-1, PMID: 30482948 PMC6430207

[41] Sanchez-Roige S Palmer AA Fontanillas P Elson SL 23andMe Research Team the Substance Use Disorder Working Group of the Psychiatric Genomics Consortium. Genome-wide association study meta-analysis of the alcohol use disorders identification test (Audit) in two population-based cohorts. Am J Psychiatry. (2019) 176:107–18. doi: 10.1176/appi.ajp.2018.18040369, PMID: 30336701 PMC6365681

[42] Clarke TK Adams MJ Davies G Howard DM Hall LS Padmanabhan S. Genome-wide association study of alcohol consumption and genetic overlap with other health-related traits in Uk biobank (N=112 117). Mol Psychiatry. (2017) 22:1376–84. doi: 10.1038/mp.2017.153, PMID: 28937693 PMC5622124

[43] Schumann G Liu C O’Reilly P Gao H Song P Xu B. Klb is associated with alcohol drinking, and its gene product beta-klotho is necessary for Fgf21 regulation of alcohol preference. Proc Natl Acad Sci U S A. (2016) 113:14372–7. doi: 10.1073/pnas.1611243113, PMID: 27911795 PMC5167198

[44] Martin J Khramtsova EA Goleva SB Blokland GAM Traglia M Walters RK. Examining sex-differentiated genetic effects across neuropsychiatric and behavioral traits. Biol Psychiatry. (2021) 89:1127–37. doi: 10.1016/j.biopsych.2020.12.024, PMID: 33648717 PMC8163257

[45] Mbatchou J Barnard L Backman J Marcketta A Kosmicki JA Ziyatdinov A. Computationally efficient whole-genome regression for quantitative and binary traits. Nat Genet. (2021) 53:1097–103. doi: 10.1038/s41588-021-00870-7, PMID: 34017140

[46] Bulik-Sullivan B Finucane HK Anttila V Gusev A Day FR Loh PR. An atlas of genetic correlations across human diseases and traits. Nat Genet. (2015) 47:1236–41. doi: 10.1038/ng.3406, PMID: 26414676 PMC4797329

[47] Bulik-Sullivan BK Loh PR Finucane HK Ripke S Yang J Schizophrenia Working Group of the Psychiatric Genomics C. Ld score regression distinguishes confounding from polygenicity in genome-wide association studies. Nat Genet. (2015) 47:291–5. doi: 10.1038/ng.3211, PMID: 25642630 PMC4495769

[48] Manolio TA Collins FS Cox NJ Goldstein DB Hindorff LA Hunter DJ. Finding the missing heritability of complex diseases. Nature. (2009) 461:747–53. doi: 10.1038/nature08494, PMID: 19812666 PMC2831613

[49] Visscher PM Hill WG Wray NR. Heritability in the genomics era–concepts and misconceptions. Nat Rev Genet. (2008) 9:255–66. doi: 10.1038/nrg2322, PMID: 18319743

[50] Ho AM Geske JR Bakalkin G Winham SJ Karpyak VM. Correlations between sex-related hormones, alcohol dependence and alcohol craving. Drug Alcohol Depend. (2019) 197:183–90. doi: 10.1016/j.drugalcdep.2019.01.029, PMID: 30840924

[51] Erol A Ho AM Winham SJ Karpyak VM. Sex hormones in alcohol consumption: A systematic review of evidence. Addict Biol. (2019) 24:157–69. doi: 10.1111/adb.12589, PMID: 29280252 PMC6585852

[52] Elgart M Goodman MO Isasi C Chen H Morrison AC de Vries PS. Correlations between complex human phenotypes vary by genetic background, gender, and environment. Cell Rep Med. (2022) 3:100844. doi: 10.1016/j.xcrm.2022.100844, PMID: 36513073 PMC9797952

[53] Simo R Saez-Lopez C Barbosa-Desongles A Hernandez C Selva DM. Novel insights in shbg regulation and clinical implications. Trends Endocrinol Metab. (2015) 26:376–83. doi: 10.1016/j.tem.2015.05.001, PMID: 26044465

[54] Hautanen A. Synthesis and regulation of sex hormone-binding globulin in obesity. Int J Obes Relat Metab Disord. (2000) 24 Suppl 2:S64–70. doi: 10.1038/sj.ijo.0801281, PMID: 10997612

[55] Carter AR Borges MC Benn M Tybjaerg-Hansen A Davey Smith G Nordestgaard BG. Combined association of body mass index and alcohol consumption with biomarkers for liver injury and incidence of liver disease: A Mendelian randomization study. JAMA Netw Open. (2019) 2:e190305. doi: 10.1001/jamanetworkopen.2019.0305, PMID: 30848805 PMC6484655

[56] Traversy G Chaput JP. Alcohol consumption and obesity: an update. Curr Obes Rep. (2015) 4:122–30. doi: 10.1007/s13679-014-0129-4, PMID: 25741455 PMC4338356

[57] Day FR Loh PR Scott RA Ong KK Perry JR. A robust example of collider bias in a genetic association study. Am J Hum Genet. (2016) 98:392–3. doi: 10.1016/j.ajhg.2015.12.019, PMID: 26849114 PMC4746366

[58] Haas CB Hsu L Lampe JW Wernli KJ Lindstrom S. Cross-ancestry genome-wide association studies of sex hormone concentrations in pre- and postmenopausal women. Endocrinology. (2022) 163(4):bqac020. doi: 10.1210/endocr/bqac020, PMID: 35192695 PMC8962449

[59] Fry A Littlejohns TJ Sudlow C Doherty N Adamska L Sprosen T. Comparison of sociodemographic and health-related characteristics of uk biobank participants with those of the general population. Am J Epidemiol. (2017) 186:1026–34. doi: 10.1093/aje/kwx246, PMID: 28641372 PMC5860371

[60] Sudlow C Gallacher J Allen N Beral V Burton P Danesh J. Uk biobank: an open access resource for identifying the causes of a wide range of complex diseases of middle and old age. PLoS Med. (2015) 12:e1001779. doi: 10.1371/journal.pmed.1001779, PMID: 25826379 PMC4380465

[61] Allen NE Arnold M Parish S Hill M Sheard S Callen H. Approaches to minimising the epidemiological impact of sources of systematic and random variation that may affect biochemistry assay data in Uk biobank. Wellcome Open Res. (2020) 5:222. doi: 10.12688/wellcomeopenres.16171.2, PMID: 33364437 PMC7739095

[62] Sun L Wang Z Lu T Manolio TA Paterson AD. Exclusionary: 10 years later, where are the sex chromosomes in Gwass? Am J Hum Genet. (2023) 110:903–12. doi: 10.1016/j.ajhg.2023.04.009, PMID: 37267899 PMC10257007

[63] Khramtsova EA Wilson MA Martin J Winham SJ He KY Davis LK. Quality control and analytic best practices for testing genetic models of sex differences in large populations. Cell. (2023) 186:2044–61. doi: 10.1016/j.cell.2023.04.014, PMID: 37172561 PMC10266536

[64] Traglia M Bseiso D Gusev A Adviento B Park DS Mefford JA. Genetic mechanisms leading to sex differences across common diseases and anthropometric traits. Genetics. (2017) 205:979–92. doi: 10.1534/genetics.116.193623, PMID: 27974502 PMC5289864

[65] Icick R Shadrin A Holen B Karadag N Parker N O’Connell K. Identification of Novel Loci and Cross-Disorder Pleiotropy through Multi-Ancestry Genome-Wide Analysis of Alcohol Use Disorder in over One Million Individuals. Res Sq. (2023) 22:rs.3.rs–3755915. doi: 10.21203/rs.3.rs-3755915/v1, PMID: 40322774 PMC12048032

[66] Zhou H Kember RL Deak JD Xu H Toikumo S Yuan K. Multi-ancestry study of the genetics of problematic alcohol use in over 1 million individuals. Nat Med. (2023) 29:3184–92. doi: 10.1038/s41591-023-02653-5, PMID: 38062264 PMC10719093

[67] Werme J van der Sluis S Posthuma D de Leeuw CA. An integrated framework for local genetic correlation analysis. Nat Genet. (2022) 54:274–82. doi: 10.1038/s41588-022-01017-y, PMID: 35288712

[68] Ning Z Pawitan Y Shen X. High-definition likelihood inference of genetic correlations across human complex traits. Nat Genet. (2020) 52:859–64. doi: 10.1038/s41588-020-0653-y, PMID: 32601477

[69] Zhang Y Lu Q Ye Y Huang K Liu W Wu Y. Supergnova: local genetic correlation analysis reveals heterogeneous etiologic sharing of complex traits. Genome Biol. (2021) 22:262. doi: 10.1186/s13059-021-02478-w, PMID: 34493297 PMC8422619

[70] Frei O Holland D Smeland OB Shadrin AA Fan CC Maeland S. Bivariate causal mixture model quantifies polygenic overlap between complex traits beyond genetic correlation. Nat Commun. (2019) 10:2417. doi: 10.1038/s41467-019-10310-0, PMID: 31160569 PMC6547727

[71] Lu Q Li B Ou D Erlendsdottir M Powles RL Jiang T. A powerful approach to estimating annotation-stratified genetic covariance via Gwas summary statistics. Am J Hum Genet. (2017) 101:939–64. doi: 10.1016/j.ajhg.2017.11.001, PMID: 29220677 PMC5812911

[72] Brown BC Asian Genetic Epidemiology Network Type 2 Diabetes C Ye CJ Price AL Zaitlen N. Transethnic genetic-correlation estimates from summary statistics. Am J Hum Genet. (2016) 99:76–88. doi: 10.1016/j.ajhg.2016.05.001, PMID: 27321947 PMC5005434

