# Supplementary material for: Genetic correlations of alcohol consumption and alcohol use disorder with sex hormone levels in females and males
Source: Front Psychiatry. 2025 Jul 22;16:1589688. doi: 10.3389/fpsyt.2025.1589688 (PMC12322815; doi:10.3389/fpsyt.2025.1589688)
Supplement: Supplementary file 1 [file DataSheet1.zip › Data Sheet 1/Supplementary Methods.docx]

**SUPPLEMENT FOR:

TITLE**

Genetic correlations of alcohol-use traits with steroid sex hormones and binding proteins in females and males

**AUTHORS**

T. Cameron Waller^1^; Ada M.-C. Ho^2^; Anthony Batzler^1^; Jennifer R. Geske^1^; Victor M. Karpyak^2^;

Joanna M. Biernacka^1,2^; Stacey J. Winham^1^

**Affiliations**

^1^Department of Quantitative Health Sciences; Mayo Clinic in Rochester, Minnesota

^2^Department of Psychiatry and Psychology; Mayo Clinic in Rochester, Minnesota

**METHODS**

**Selection and preparation of summary statistics for GWAS on alcohol-use and sex-hormone traits**

We selected GWAS summary statistics that would be suitable to estimate the genetic correlations of alcohol-use traits with levels of sex-hormones and their binding proteins. To identify relevant published and publicly available GWAS, we searched PubMed (https://pubmed.ncbi.nlm.nih.gov/) and the NHGRI-EBI GWAS Catalog (https://www.ebi.ac.uk/gwas/) for studies using the terms “alcohol use”, “alcohol consumption”, “alcohol dependence”, “alcohol use disorder”, “sex hormones”, “testosterone”, “estradiol”, “sex hormone binding globulin”, and “albumin”; we further searched for additional sex hormones such as “progesterone”, “follicle stimulating hormone”, and “luteinizing hormone”, but did not identify any studies of sufficient sample size. We prioritized studies with the largest samples sizes, along with clinically relevant trait definitions. For example, we prioritized the alcohol-related traits of alcohol dependence (1) and alcohol consumption (2) over the less clinically specific trait of problematic alcohol use (3). The GWAS summary statistics for AUD (1) and Alcohol Consumption (2) were retrieved from the Psychiatric Genomics Consortium (https://pgc.unc.edu/) and from the University of Minnesota data repository for GSCAN (https://genome.psych.umn.edu/index.php/GSCAN), respectively. The GWAS summary statistics for Testosterone, Bioavailable Testosterone, and SHBG (4) were retrieved from the GWAS Catalog. The GWAS summary statistics for Estradiol (5) retrieved from the Zenodo archive (https://zenodo.org). The GWAS summary statistics for albumin (6) was retrieved from the GWAS catalog. The referenced publication (including PMID) and name of the retrieved data file are shown in Table S1.

The prior GWAS of levels of steroid sex hormones and their binding proteins (Table S1) were based on UK Biobank data. While the LDSC method appears to be reasonably robust in comparisons of GWAS from overlapping sample cohorts (7), we took extra precaution to avoid the risk of bias or inflation of type I errors by using summary statistics from GWAS of alcohol consumption (2) and alcohol dependence (1) based on sample cohorts that did not include UK Biobank data. These specific versions of summary statistics from GWAS of alcohol consumption and alcohol dependence were not identical to the primary discovery GWAS described in the respective publications.

Before use in our study, we harmonized each set of GWAS summary statistics in our collection (Table S1). A few sets of GWAS summary statistics were missing information that was relevant to our subsequent analyses, such as the total sample size of the GWAS cohort or the counts of cases and controls in GWAS of dichotomous traits, and we filled this missing information by consulting the respective publications and accompanying documentation. For summary statistics from GWAS of dichotomous traits, namely risk of alcohol dependence (1) and detectability of estradiol levels (5), we also calculated the effective sample size (N_effective_) (8) for use in our subsequent analyses. Where necessary, we used CrossMap (9) with chain files from Ensembl (10) to convert genomic coordinates to the GRCh37 assembly of the human genome. After these preliminary steps, we applied GWAS2VCF (11) (https://github.com/MRCIEU/gwas2vcf) to clean and harmonize all GWAS summary statistics in our collection. This tool verified accurate reference SNP cluster identifiers (rsIDs) of each variant locus by comparison to build 155 of dbSNP (RefSeq accession “GCF_000001405.25”), and it also flipped designations of reference or alternate (effect) alleles and adjusted effect directions and allele frequencies accordingly to align all GWAS summary statistics to the same reference human genome sequence (file: “human_g1k_v37.fasta.gz”; human genome assembly GRCh37).

**Estimates of SNP heritability and genetic correlation**

We used version v1.0.0 of LDSC (12, 13) (https://github.com/bulik/ldsc) to munge GWAS summary statistics and to estimate SNP heritability (h^2^) and genetic correlation (r_g_). To ensure the use of SNPs with high-confidence quality of genotype imputation, we filtered GWAS summary statistics to SNPs (file: “w_hm3.snplist”) from the HapMap3 (14) that had minor allele frequency (MAF) greater than 5% in the European-ancestry cohort from Phase 1 of the 1000 Genomes Project (15). We also used reference LD scores (directory: “eur_w_ld_chr”) calculated on the European-ancestry cohort from Phase 1 of the 1000 Genomes Project (15). For LDSC estimates of SNP heritability and genetic correlation, we did not constrain the regression intercept to decrease the potential for bias from LD differences between reference and sample populations or from cryptic sample overlap (12, 13). For GWAS of dichotomous traits, we estimated SNP heritability on the liability scale using the values of sample and population prevalence in Table S1, and we estimated SNP heritability on the observed scale for all other GWAS of continuous traits. All estimates of genetic correlation were based on 920,164 or more SNPs shared between the two sets of GWAS summary statistics (Table 1, Table 2).

**RESULTS**

**SNP heritability of GWAS on alcohol-use and sex-hormone traits used for genetic correlation**

Based on the prior-published GWAS summary statistics that we used to estimate genetic correlations, Table S1 presents the SNP heritability estimates due to common genetic variants for quantity of alcohol consumption (2), risk of alcohol dependence (1), and blood levels of total testosterone (4), bioavailable testosterone (4), estradiol (5), SHBG (4), and albumin (6). The SNP heritability estimate was low for alcohol consumption (h^2^ = 0.0397, se = 0.0030) but was higher for alcohol dependence (h^2^ = 0.1021, se = 0.0166). The SNP heritability for testosterone was higher, with estimates for total testosterone of 0.1621 (se = 0.0106) in females and 0.2133 (se = 0.0309) in males; findings for bioavailable testosterone were similar for females (h^2^ = 0.1668, se = 0.0167) but considerably lower for males (h^2^ = 0.1238, se = 0.0081). In contrast, the SNP heritability estimates for detectable levels of estradiol were much lower in females (h^2^ = 0.0170, se = 0.0069) than in males (h^2^ = 0.1075, se = 0.0231). For SHBG the estimates of SNP heritability were higher, particularly with adjustment for BMI (Table S1), in both females (h^2^ = 0.3027, se = 0.0320) and males (h^2^ = 0.3151, se = 0.0386). Finally, the SNP heritability for albumin in a sex-combined cohort was 0.1303 (se = 0.0078).

**References**

1. Walters RK, Polimanti R, Johnson EC, McClintick JN, Adams MJ, Adkins AE, et al. Transancestral Gwas of Alcohol Dependence Reveals Common Genetic Underpinnings with Psychiatric Disorders. *Nat Neurosci* (2018) 21(12):1656-69. Epub 20181126. doi: 10.1038/s41593-018-0275-1.

2. Liu M, Jiang Y, Wedow R, Li Y, Brazel DM, Chen F, et al. Association Studies of up to 1.2 Million Individuals Yield New Insights into the Genetic Etiology of Tobacco and Alcohol Use. *Nat Genet* (2019) 51(2):237-44. Epub 20190114. doi: 10.1038/s41588-018-0307-5.

3. Zhou H, Sealock JM, Sanchez-Roige S, Clarke TK, Levey DF, Cheng Z, et al. Genome-Wide Meta-Analysis of Problematic Alcohol Use in 435,563 Individuals Yields Insights into Biology and Relationships with Other Traits. *Nat Neurosci* (2020) 23(7):809-18. Epub 20200525. doi: 10.1038/s41593-020-0643-5.

4. Ruth KS, Day FR, Tyrrell J, Thompson DJ, Wood AR, Mahajan A, et al. Using Human Genetics to Understand the Disease Impacts of Testosterone in Men and Women. *Nat Med* (2020) 26(2):252-8. Epub 20200210. doi: 10.1038/s41591-020-0751-5.

5. Schmitz D, Ek WE, Berggren E, Hoglund J, Karlsson T, Johansson A. Genome-Wide Association Study of Estradiol Levels and the Causal Effect of Estradiol on Bone Mineral Density. *J Clin Endocrinol Metab* (2021) 106(11):e4471-e86. doi: 10.1210/clinem/dgab507.

6. Mbatchou J, Barnard L, Backman J, Marcketta A, Kosmicki JA, Ziyatdinov A, et al. Computationally Efficient Whole-Genome Regression for Quantitative and Binary Traits. *Nat Genet* (2021) 53(7):1097-103. Epub 20210520. doi: 10.1038/s41588-021-00870-7.

7. Zhang Y, Cheng Y, Jiang W, Ye Y, Lu Q, Zhao H. Comparison of Methods for Estimating Genetic Correlation between Complex Traits Using Gwas Summary Statistics. *Brief Bioinform* (2021) 22(5). doi: 10.1093/bib/bbaa442.

8. Willer CJ, Li Y, Abecasis GR. Metal: Fast and Efficient Meta-Analysis of Genomewide Association Scans. *Bioinformatics* (2010) 26(17):2190-1. Epub 20100708. doi: 10.1093/bioinformatics/btq340.

9. Zhao H, Sun Z, Wang J, Huang H, Kocher JP, Wang L. Crossmap: A Versatile Tool for Coordinate Conversion between Genome Assemblies. *Bioinformatics* (2014) 30(7):1006-7. Epub 20131218. doi: 10.1093/bioinformatics/btt730.

10. Cunningham F, Allen JE, Allen J, Alvarez-Jarreta J, Amode MR, Armean IM, et al. Ensembl 2022. *Nucleic Acids Res* (2022) 50(D1):D988-D95. doi: 10.1093/nar/gkab1049.

11. Lyon MS, Andrews SJ, Elsworth B, Gaunt TR, Hemani G, Marcora E. The Variant Call Format Provides Efficient and Robust Storage of Gwas Summary Statistics. *Genome Biol* (2021) 22(1):32. Epub 20210113. doi: 10.1186/s13059-020-02248-0.

12. Bulik-Sullivan BK, Loh PR, Finucane HK, Ripke S, Yang J, Schizophrenia Working Group of the Psychiatric Genomics C, et al. Ld Score Regression Distinguishes Confounding from Polygenicity in Genome-Wide Association Studies. *Nat Genet* (2015) 47(3):291-5. Epub 20150202. doi: 10.1038/ng.3211.

13. Bulik-Sullivan B, Finucane HK, Anttila V, Gusev A, Day FR, Loh PR, et al. An Atlas of Genetic Correlations across Human Diseases and Traits. *Nat Genet* (2015) 47(11):1236-41. Epub 20150928. doi: 10.1038/ng.3406.

14. International HapMap C, Altshuler DM, Gibbs RA, Peltonen L, Altshuler DM, Gibbs RA, et al. Integrating Common and Rare Genetic Variation in Diverse Human Populations. *Nature* (2010) 467(7311):52-8. doi: 10.1038/nature09298.

15. Genomes Project C, Abecasis GR, Auton A, Brooks LD, DePristo MA, Durbin RM, et al. An Integrated Map of Genetic Variation from 1,092 Human Genomes. *Nature* (2012) 491(7422):56-65. doi: 10.1038/nature11632.
